# Evaluating the Effect of a Web-Based E-Learning Tool for Health Professional Education on Clinical Vancomycin Use: Comparative Study

**DOI:** 10.2196/mededu.7719

**Published:** 2018-02-26

**Authors:** Stuart Evan Bond, Shelley P Crowther, Suman Adhikari, Adriana J Chubaty, Ping Yu, Jay P Borchard, Craig Steven Boutlis, Wilfred Winston Yeo, Spiros Miyakis

**Affiliations:** ^1^ Department of Pharmacy Wollongong Hospital Illawarra Shoalhaven Local Health District Wollongong Australia; ^2^ School of Medicine Faculty of Science, Medicine and Health University of Wollongong Wollongong Australia; ^3^ Illawarra Health and Medical Research Institute University of Wollongong Wollongong Australia; ^4^ Pinderfields Hospital Mid Yorkshire Hospitals NHS Trust Wakefield United Kingdom; ^5^ Department of Pharmacy St George Hospital South Eastern Sydney Local Health District Kogarah Australia; ^6^ St George Clinical School Faculty of Medicine University of New South Wales Kogarah Australia; ^7^ Department of Pharmacy Prince of Wales Hospital South Eastern Sydney Local Health District Randwick Australia; ^8^ School of Computing and Information Technology Faculty of Engineering and Information Sciences University of Wollongong Wollongong Australia; ^9^ Research Central Wollongong Hospital Illawarra Shoalhaven Local Health District Wollongong Australia; ^10^ Department of Infectious Diseases Wollongong Hospital Illawarra Shoalhaven Local Health District Wollongong Australia; ^11^ Division of Medicine Wollongong Hospital Illawarra Shoalhaven Local Health District Wollongong Australia

**Keywords:** nursing education, pharmacy education, medical education, continuing education, survey methods, antibacterial agents

## Abstract

**Background:**

Internet-based learning for health professional education is increasing. It offers advantages over traditional learning approaches, as it enables learning to be completed at a time convenient to the user and improves access where facilities are geographically disparate. We developed and implemented the Vancomycin Interactive (VI) e-learning tool to improve knowledge on the clinical use of the antibiotic vancomycin, which is commonly used for treatment of infections caused by methicillin-resistant Staphylococcus aureus (MRSA).

**Objective:**

The aims of this study were to evaluate the effect of the VI e-learning tool on (1) survey knowledge scores and (2) clinical use of vancomycin among health professionals.

**Methods:**

We conducted a comparative pre-post intervention study across the 14 hospitals of two health districts in New South Wales, Australia. A knowledge survey was completed by nurses, doctors, and pharmacists before and after release of a Web-based e-learning tool. Survey scores were compared with those obtained following traditional education in the form of an email intervention. Survey questions related to dosing, administration, and monitoring of vancomycin. Outcome measures were survey knowledge scores among the three health professional groups, vancomycin plasma trough levels, and vancomycin approvals recorded on a computerized clinical decision support system.

**Results:**

Survey response rates were low at 26.87% (577/2147) preintervention and 8.24% (177/2147) postintervention. The VI was associated with an increase in knowledge scores (maximum score=5) among nurses (median 2, IQR 1-2 to median 2, IQR 1-3; *P*<.001), but not among other professional groups. The comparator email intervention was associated with an increase in knowledge scores among doctors (median 3, IQR 2-4 to median 4, IQR 2-4; *P*=.04). Participants who referred to Web-based resources while completing the e-learning tool achieved higher overall scores than those who did not (*P*<.001). The e-learning tool was not shown to be significantly more effective than the comparator email in the clinical use of vancomycin, as measured by plasma levels within the therapeutic range.

**Conclusions:**

The e-learning tool was associated with improved knowledge scores among nurses, whereas the comparator email was associated with improved scores among doctors. This implies that different strategies may be required for optimizing the effectiveness of education among different health professional groups. Low survey response rates limited conclusions regarding the tool’s effectiveness. Improvements to design and evaluation methodology may increase the likelihood of a demonstrable effect from e-learning tools in the future.

## Introduction

### Internet-Based Learning

Traditional face-to-face approaches to health professional education are being challenged by busy trainee schedules involving increased clinical demands and decreased available time [1,2]. These barriers can be addressed through the use of Internet-based learning (IBL) approaches, which can be completed at a time convenient to the user [3]. It may also be useful if health professional education is required across geographically disparate hospital locations. Effective IBL tools should provide entertainment and supply the user with knowledge, skills, or attitudes useful in real life [4]. Recently, there has been considerable development in novel IBL methodologies for health professional education (eg, serious games) with common topics relating to surgical skills training, critical care, and emergency triage [2,5]. Some studies showed improvements in test scores [2]; however, study design was heterogeneous and none focused on the antibiotic vancomycin as an educational target.

### Vancomycin Education

Vancomycin is the main antibiotic used for treatment of infections caused by methicillin-resistant *Staphylococcus aureus* [6]. Problems associated with vancomycin use across multiple professions include the requirement for a loading dose in serious infections, side effects when administered too rapidly, and the need to monitor vancomycin plasma levels (or concentrations) [6]. Therefore, several studies have described interventions to improve clinical use of vancomycin [7-14]. Specific topics addressed in those studies were dosing [7,9,11,14], administration [7], and therapeutic drug monitoring (TDM) [7-10,12,13]. Educational targets were nurses, doctors, or pharmacists, with one TDM study conducting multidisciplinary interventions [12]. In a previous study [15], we described the design and implementation process of a Web-based e-learning tool (Vancomycin Interactive; VI) that employed serious game design concepts including interactivity and entertainment to provide education on vancomycin. To our knowledge, this study is the first to compare outcomes of a vancomycin e-learning tool with a standard didactic email intervention.

The design and implementation methodology for the Vancomycin Interactive Web-based e-learning tool has been provided in a prior publication, “Design and Implementation of a Novel Web-Based E-Learning Tool for Education of Health Professionals on the Antibiotic Vancomycin” in the Journal of Medical Internet Research [15].

### Aims of This Study

The aims of this study were to assess the VI e-learning tool versus standard email intervention for (1) effects on health professionals’ vancomycin knowledge and (2) effects on quality of vancomycin use measured by both vancomycin plasma trough levels and approvals for use recorded on a computerized clinical decision support system (CDSS; Guidance MS, Melbourne Health [16]).

## Methods

**Figure 1:**
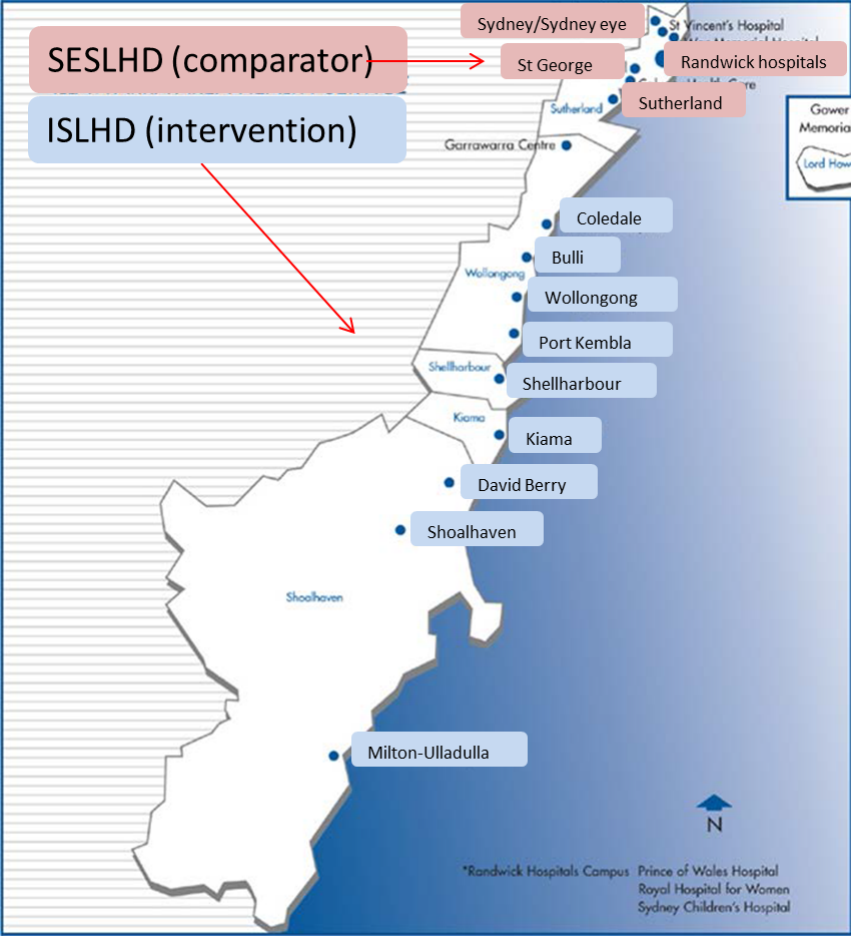
Intervention (Illawarra Shoalhaven Local Health District; ISLHD) and comparator (South Eastern Sydney Local Health District; SESLHD) sites.

This comparative pre-post intervention study took place in Illawarra Shoalhaven Local Health District (ISLHD; Vancomycin Interactive intervention site, 1000 total beds, 700 acute beds) and South Eastern Sydney Local Health District (SESLHD; comparator email intervention site, 1500 total beds, 1200 acute beds), located in New South Wales, Australia (Figure 1). These health districts cover a geographic area of 6331 km^2^ and have an estimated population of 1.17 million, reaching from central Sydney to a 3-hour drive south [17]. The districts’ 14 hospitals range from small rural facilities to large tertiary metropolitan hospitals. The comparator email site was selected due the following: a shared information technology platform with the e-learning intervention site, geographical proximity, and existing clinical and professional networks.

### Preintervention and Postintervention Vancomycin Knowledge Survey

An anonymous Web-based survey was created using Survey Monkey (SurveyMonkey Inc, Palo Alto, CA) to determine preintervention experience/confidence and knowledge of vancomycin use among nurses, doctors, and pharmacists across two health districts [15]. A 4-point Likert scale was used to determine levels of experience and confidence relating to knowledge questions on dosing, administration, and monitoring of vancomycin (see Multimedia Appendix 1).

Postintervention, a second survey with the same questions was sent to the intervention and comparator sites. User testing indicated that the preintervention survey would take approximately 2 minutes to complete and the postintervention surveys would take 3 minutes, because additional user feedback was sought on the VI and comparator email. Requests for survey participation are included as Multimedia Appendices 2 and 3. A survey question on resources used to answer the survey was also analyzed.

### Vancomycin Interactive and Clinical Email Intervention

Educational content was developed locally for the VI on dosing, administration, and TDM of vancomycin [6,18]. The learning objectives of the VI for target users (nurses, doctors, and pharmacists) were to improve knowledge of vancomycin dosing, administration, and TDM. The VI (ISLHD) [19] depicted a case study involving interaction between a patient and a health professional, both played by professional actors. The user interface consisted of video clips interspersed with interactive question and answer scenarios [15]. User testing indicated that the VI would take approximately 10 minutes to complete. An email (taking 2-3 minutes to read) with the same clinical content and learning objectives was developed as a comparator intervention (Multimedia Appendix 4). To allow for the differences in the two media, there were some minor variations in clinical content between the VI and email that related to administration of vancomycin.

Release and advertisement of the VI (email, newsletters, link on intranet home page) to the intervention site occurred on July 27, 2015. The clinical email intervention was then sent to nurses, doctors, and pharmacists at the comparator site (Multimedia Appendix 4). Following completion of the second survey, the VI website was also advertised to the comparator site. To allow for sufficient dissemination of the interventions, the postintervention survey was open from December 1, 2015 to January 31, 2016.

### Vancomycin Trough Plasma Levels and Approvals on the Clinical Decision Support System

Vancomycin plasma levels from a 4-month period before and a 2-month period after the VI and comparator email were analyzed to determine changes in the proportion of levels in the therapeutic range. The postintervention period was limited to 2 months in order to conclude before the annual intake of new junior doctors. Criteria for dose adjustment were as follows: (1) 0-9 mg/L: increase dose; (2) 10-14 mg/L: maintain or increase dose (depending on severity of infection and clinical status); (3) 15-20 mg/L: maintain current dose; (4) 20-25 mg/L: maintain or reduce dose (depending on severity of infection and clinical status); and (5) >25 mg/L: withhold dose until trough level less than 20 mg/L and seek expert advice [6]. The number of vancomycin levels as a proportion of the total number of vancomycin CDSS approvals was analyzed to determine frequency of vancomycin use. Pharmacy dispensing software did not allow for patient-level data on vancomycin dispensing to be analyzed because vancomycin was distributed as ward stock in some hospitals. Hence, vancomycin CDSS approvals were used as the best-available indicator for total vancomycin use.

### Outcome Measures

We compared total vancomycin knowledge survey scores preintervention and postintervention, within and between e-learning intervention and comparator email intervention sites. The number of vancomycin levels in the therapeutic range, the median number of vancomycin levels and ratio of vancomycin levels to CDSS vancomycin approvals between sites were also analyzed.

### Statistical Analyses

Chi-square and Fisher exact tests were used for proportions. For continuous data, normality was assessed using a skewness/kurtosis statistic [20]. A skewed distribution was denoted by *P*<.05. Kruskal-Wallis and follow-up Wilcoxon rank-sum tests were used to investigate between effects with nonnormal distributions. Multivariate analysis was performed to examine influential factors (profession, site, pre- or postintervention) on correct survey responses. Given the limited literature in this field of research, a sample size calculation was conducted based on Monte-Carlo simulations of pilot data. This calculation was performed to estimate the sample size required for the effect of site on total knowledge score. The expected distributions of knowledge scores for the intervention (mean 3.30, SD 1.47) and control sites (mean 2.85, SD .48) were derived from pilot data. These hypothesized data structures were then randomly resampled with 10,000 iterations under different sample size conditions to estimate the required sample size to detect a difference. Based on these simulations, it was calculated that a sample size of 226 in each group was required to achieve 90% power for significance of *P*<.05. Statistical analyses were performed using Stata statistical software release 14 (Statacorp LP, College Station, TX, USA).

### Ethics

Ethics approval was granted by the Joint University of Wollongong and Illawarra Shoalhaven Local Health District Health and Medical Human Research Ethics Committee (EC00150), approval number HE15/005. The VI website contained a disclaimer that anonymous data collected from the video could be used for educational and research purposes.

## Results

### Vancomycin Knowledge Survey

The response rate to the preintervention survey was 26.87% (577 responses from 2147 email recipients). The response rates by profession were 24.4% (236/967) for nurses, 25.33% (271/1070) for doctors, and 63.6% (70/110) for pharmacists (*P*<.001; previously reported [15]). Postintervention, there were 177/2147 survey responses (8.24% response rate), comprising 88 nurses, 69 doctors, and 20 pharmacists (*P*<.001).

The median knowledge survey score for nurses increased post-VI (*P*<.001; Table 1). No significant differences pre- and post-VI were observed for doctors or pharmacists. At the comparator email intervention site, the median knowledge survey score increased postintervention for doctors (*P*=.04; Table 1).

**Table 1:** Preintervention and postintervention vancomycin knowledge survey scores for the intervention site using Vancomycin Interactive and the comparator email site.

Profession	Vancomycin Interactive intervention site, median (IQR)^a^			Comparator site, median (IQR)^a^		
	Pre (n=278)	Post (n=107)	*P* value	Pre (n=299)	Post (n=70)	*P* value
Nurse	2 (1-2)	2 (1-3)	<.001	2 (1-3)	3 (2-4)	.17
Doctor	3 (2-4)	4 (2-4)	.28	3 (2-4)	4 (2-4)	.04
Pharmacist	5 (4-5)	4 (4-5)	.40	5 (4-5)	5 (4-5)	.87

^a^IQR: interquartile range; Out of a maximum of 5.

**Table 2:** Preintervention (4 months) and postintervention (2 months) vancomycin plasma trough levels for intervention and comparator sites.

Trough level (mg/L)	Vancomycin Interactive intervention site			Comparator email site		
	Pre (n=429)	Post (n=151)	*P* value	Pre (n=1571)	Post (n=316)	*P* value
0-9 (subtherapeutic), n (%)	48 (11)	17 (11)	.98	259 (16)	50 (16)	.77
10-14 (low therapeutic), n (%)	91 (21)	28 (19)	.49	362 (23)	62 (20)	.18
15-20 (therapeutic), n (%)	168 (39)	54 (35)	.46	515 (33)	98 (31)	.54
21-25 (high therapeutic), n (%)	72 (17)	36 (24)	.06	260 (17)	66 (21)	.06
>25 (supratherapeutic), n (%)	50 (12)	16 (11)	.73	175 (11)	40 (13)	.44
Median (IQR^a^)	18 (13-21)	17 (13-22)	.62	16 (12-21)	17 (12-22)	.14

^a^IQR: interquartile range.

### Resources Used to Answer Survey Questions

To the question, “Did you refer to any resources to answer these questions?” 595 of 754 (78.9%) participants responded “no.” Of those 595, 424 (71.3%) self-reported that they guessed some or all of the answers, whereas 171 (28.7%) reported that they knew the answers. The remaining 159 of 754 (21%) respondents self-reported that they referred to resources for answering the questions. The resources quoted were local guidelines (49/159, 30.9%) and the *Australian Medicines Handbook* or *Therapeutic Guidelines: Antibiotic* (110/159, 69.1%).

### Multivariate Analysis of Knowledge Survey Scores

Several factors were associated with an increased knowledge survey score. Compared to nurses, pharmacists (regression coefficient 1.93, 95% CI 1.63-2.23; *P*<.001) and doctors (regression coefficient 0.89, 95% CI 0.70-1.09; *P*<.001) had increased likelihood of a higher survey score. Postintervention survey participation was also associated with a higher score (regression coefficient 0.41, 95% CI 0.20-0.62; *P*<.001) than preintervention. Referring to online resources was associated with a higher score compared with responses that participants self-reported that they knew or guessed the answers (regression coefficient 0.98, 95% CI 0.75-1.20; *P*<.001). The comparator site was not significantly associated with increased likelihood of higher survey scores (regression coefficient 0.16, 95% CI –0.02 to 0.34; *P*=.08).

### Vancomycin Therapeutic Drug Monitoring

From January 1 to April 30, 2015, there were 429 vancomycin trough plasma levels taken at the intervention site (ISLHD) and 1571 levels for the comparator site (SESLHD). During the postintervention period of December 1, 2015 to January 31, 2016, there were 151 levels reported at the intervention site and 316 levels at the comparator site. As shown in Table 2, there were no significant postintervention differences in the proportion of vancomycin levels in the subtherapeutic (0-9 mg/L), therapeutic (10-14, 15-20, and 21-25 mg/L), or supratherapeutic (>25 mg/L) ranges. There were increases in the number of levels in the high therapeutic range (20-25 mg/L) at both sites; however, those differences did not reach statistical significance. There were no significant pre-post intervention differences in median vancomycin levels at the intervention site or comparator site (Table 2).

### Vancomycin Trough Plasma Levels Compared With Vancomycin Clinical Decision Support System Approvals

The proportion of vancomycin trough levels to vancomycin CDSS approvals at the intervention site decreased from 429/399 preintervention (1.1 levels for every vancomycin approval) to 151/196 postintervention (0.8 levels/approval). At the comparator site, the proportion of vancomycin levels to vancomycin CDSS approvals decreased from 1571/399 preintervention (3.9 levels/approval) to 314/199 postintervention (1.6 levels/approval).

## Discussion

### Principal Findings

This study compared the educational effect of an interactive Web-based e-learning tool with a comparator email intervention. Two different learning modalities were investigated among three different health professional groups. The e-learning tool was associated with improved survey scores among nurses, whereas the comparator email intervention was associated with improved scores among doctors. Not unexpectedly, pharmacists and doctors had higher overall knowledge scores than nurses due to the greater number of questions considered relevant to those groups. Also, participants who referred to Web-based resources while completing the survey had higher survey scores than those who did not.

Concerningly, only approximately one-third of preintervention and postintervention vancomycin levels taken at both sites fell within the recommended therapeutic range of 15-20 mg/L. This figure rose to 73% when the ranges 10-14 mg/L, 15-20mg/L, and 21-25 mg/L were combined, which includes all potential recommended therapeutic ranges [6]. The proportion of vancomycin levels to CDSS approvals decreased at both sites, perhaps signifying a reduction in the ordering of unnecessary levels or shorter vancomycin courses requiring fewer levels. A greater proportion of levels/approvals was observed at the email intervention site in both preintervention and postintervention phases, which may have resulted from differences in acuity between sites.

In previous studies, strategies for improving the clinical use of vancomycin have included use of loading doses [14], implementation of guidelines [9], education [7,10,13], and CDSSs [8,11,12]. None of those educational interventions incorporated a Web-based e-learning tool, and the predominant methodology was uncontrolled pre-post intervention at single hospital sites. One study reported development of a serious game to improve general antimicrobial prescribing, but it did not focus on vancomycin [21]. A review of educational games for health professionals emphasized the need for more research with improved study methodology [22]. Our study differed in its multisite approach, comparison of an e-learning tool with a standard email intervention, and targeting of multiple health professional groups.

### Interpretation of Results

The difference in efficacy between the VI (improved nurses’ scores) and the email (improved doctors’ scores) may have arisen from nurses’ increased familiarity and engagement with online learning modules, whereas for doctors a didactic learning style may be more suitable. Additionally, the short time to read a clinical email may have been more convenient for doctors. Referring to resources was associated with improved survey scores, which emphasizes the importance of guideline access in the clinical setting. Some aspects of our study design may be applicable to facilities where there are geographic barriers to use of face-to-face education, such as rural and regional hospitals. Potential improvements to the structure of the VI through greater application of serious game methodology include more interactivity, scoring, and competition [23,24]. Those features could result in a greater level of user acceptance and effectiveness.

### Study Limitations

The total number of vancomycin levels at the comparator site was considerably higher than at the intervention site, which may be due to differences in case mix (number of acute beds), antimicrobial use, and background educational culture. However, the proportion of satisfactory levels (ie, those in therapeutic range) did not differ between the sites. Furthermore, similar sizeable reductions in the number of vancomycin levels ordered were experienced at both sites. Some of this reduction may have been associated with seasonal variation of vancomycin use, although unlike other antibiotics, vancomycin is not typically associated with strong seasonal variation [25]. The low response rate to the postintervention survey limited the power of pre-post intervention comparisons; however, 78% of the desirable sample size was reached. Potential reasons for this reduction include the perception of staff that the postintervention survey was the same as the preintervention survey, despite clarifications that were provided in the email title and text, and appropriate advertisement in staff newsletters. The validity of the findings is supported by similar proportions of different health professionals in the two time periods. In addition, the denominator included all targeted health professionals including those not involved in the day-to-day clinical use of vancomycin, which is likely to have reduced the response rate.

The higher scores from the postintervention survey may have resulted from participant bias (ie, only more experienced and enthusiastic staff may have responded to the second survey). Time-dependent bias may also have influenced some of the improvement in survey scores, whereby increased time in a clinical role may have resulted in greater knowledge of vancomycin use over the study period. A crossover design might have partially alleviated this factor, but this was not possible in our case due to the rotation of junior doctors between the two sites. Absence of a code to allow matching of individual responses may also have limited conclusions about the effect of the interventions on knowledge level.

Pooled presurvey results were compared with pooled postsurvey results resulting in a dataset that included both independent and dependent data. Although unavoidable according to the study design, inclusion of dependent data increased the risk of type 1 error. Additionally, pooling of the survey response data when there were differences between health professional groups may have limited conclusions on pre-post differences. Although individual predictors in the multivariate regression were likely to be skewed, the normality of the error between observed and predicted values was of primary interest in this study.

There were some minor variations in clinical content between the VI and email; however, they related only to administration of vancomycin and references used for development of content were the same for both interventions. Participants who referred to guidelines while completing the survey attained higher scores than those who did not. Although this was unavoidable in a pragmatic study, it was still a desirable outcome because those participants were using recommended national or local guidelines. The time to complete the e-learning tool (10 minutes) was longer than the email intervention (2-3 minutes); the duration of the email may have been more appropriate for doctors in a busy clinical context and this has likely contributed to the low response rates. As reported in our previous study [15], there was low uptake of the VI during the study period and we did not measure the number of comparator emails read by staff. There may have been some word-of-mouth leakage of the VI to the comparator site; however, study data collection was completed before the junior doctor rotation. Given the use of paper medications charts, the number of CDSS approvals was used as a surrogate for vancomycin prescribing. Investigation of effects of the educational tools on clinical practice was beyond the scope of this study. We did not examine quality measures of vancomycin use, such as time to first therapeutic level, levels obtained at steady state, or clinical outcomes associated with the intervention; further research aims to examine these effects. Linkage of survey-participant responses was desirable, but was not achievable within the ethical requirement for an anonymous survey. The timeframe for postintervention data collection was a relatively short 2 months, which may not have been long enough for transfer of knowledge into practice. Addressing some of these limitations may improve the likelihood of demonstrating significant effects from an e-learning tool.

### Conclusions

Different health professional groups can be educated by using different targeted learning modalities. Significant challenges can be experienced during design and evaluation of comparative e-learning research. Further studies should aim to improve structural elements of e-learning tools and enhance evaluation, including clinical outcomes, through an approach governed by a newly proposed checklist. The impact of continuous e-learning education on clinical practice needs to be assessed continuously for a long period of time.

## Appendix

Multimedia Appendix 1 Preintervention vancomycin knowledge survey.

Multimedia Appendix 2 Preintervention survey request emailed to staff at intervention/comparator sites.

Multimedia Appendix 3 Postintervention survey request emailed to staff at intervention/comparator sites.

Multimedia Appendix 4 Clinical email update sent to staff at the comparator sites.

## Abbreviations

CDSS: clinical decision support system
IBL: Internet-based learning
ISLHD: Illawarra Shoalhaven Local Health District
SESLHD: South Eastern Sydney Local Health District
TDM: therapeutic drug monitoring
