# Supplementary material for: Evaluating the Effect of a Web-Based E-Learning Tool for Health Professional Education on Clinical Vancomycin Use: Comparative Study
Source: JMIR Med Educ. 2018 Feb 26;4(1):e5. doi: 10.2196/mededu.7719 (PMC5847818; doi:10.2196/mededu.7719)
Supplement: Multimedia Appendix 1 [file mededu_v4i1e5_app1.pdf]

Multimedia Appendix 1. Pre-intervention vancomycin knowledge survey [1]

1. What is your profession?
   a. Nurse
   b. Doctor (intern, resident, registrar, consultant)
   c. pharmacist
2. How much experience do you have with calculating doses of vancomycin?
   a. None at all
   b. Only a little
   c. I am reasonably experienced
   d. I am very experienced
3. What do you think is the correct loading dose for vancomycin?
   a. 5-15mg/kg
   b. 10-20mg/kg
   c. 15-18mg/kg
   d. 25-30mg/kg
4. What is an appropriate maintenance dose for vancomycin for a patient with a

   creatinine clearance of greater than 90mL/min?
   a. 1.5g 12-hourly
   b. 1g 12-hourly
   c. 1g 48-hourly
   d. 2.5g 12-hourly
5. How confident are you to determine the administration rate for vancomycin?
   a. Not at all
   b. Only a little
   c. I am reasonably confident
   d. I am very confident
6. At what rate should vancomycin be administered to avoid red man syndrome?
   a. 1mg/min
   b. 10mg/min
   c. 50mg/min
   d. 100mg/min
7. How confident are you to provide advice on vancomycin monitoring?
   a. Not at all
   b. Only a little
   c. Reasonably
   d. Very
8. When should the first level be taken for a dose of 1g 12hrly?
   a. Within 24 hours
   b. Within a week
   c. Before the fourth dose
   d. Before the third dose
9. What is the usual target range for vancomycin plasma trough levels?
   a. 1-2mg/L
   b. 5-10mg/L

c. 15-20mg/L
d. 40-45mg/L

10. Did you refer to any resources to answer these questions?
    a. Yes – Therapeutic Guidelines or Australian Medicines Handbook
    b. Yes – Local guidelines
    c. No – I guessed the answers
    d. No – I knew the answers
