# Supplementary material for: Evaluating the Effect of a Web-Based E-Learning Tool for Health Professional Education on Clinical Vancomycin Use: Comparative Study
Source: JMIR Med Educ. 2018 Feb 26;4(1):e5. doi: 10.2196/mededu.7719 (PMC5847818; doi:10.2196/mededu.7719)
Supplement: Multimedia Appendix 2 [file mededu_v4i1e5_app2.pdf]

Multimedia Appendix 2. Pre-intervention survey request emailed to staff at intervention/comparator sites

“Dear health professional (nurse, doctor, pharmacist),

Please take two minutes to complete a brief knowledge survey on the dosing, administration and monitoring of vancomycin.

This survey will help us to develop a vancomycin learning module targeted to your needs at SESLHD and ISLHD Hospitals.

https://www.surveymonkey.com/s/vancomycin

We would be grateful for your time to answer 10 quick questions.

Thank you”
