# Supplementary material for: Evaluating the Effect of a Web-Based E-Learning Tool for Health Professional Education on Clinical Vancomycin Use: Comparative Study
Source: JMIR Med Educ. 2018 Feb 26;4(1):e5. doi: 10.2196/mededu.7719 (PMC5847818; doi:10.2196/mededu.7719)
Supplement: Multimedia Appendix 3 [file mededu_v4i1e5_app3.pdf]

Multimedia Appendix 3. Post-intervention survey request emailed to staff at intervention/ comparator sites

Dear colleague,

Please take 3 minutes to complete this second survey on the anti-MRSA antibiotic vancomycin. We would love to know if the VI/vancomycin email update was useful, and how we can improve antibiotic education in the future. Answers will remain confidential.

To complete the survey please click: https://www.surveymonkey.com/r/vancomycin

Thanks!”
