# Supplementary material for: Evaluating the Effect of a Web-Based E-Learning Tool for Health Professional Education on Clinical Vancomycin Use: Comparative Study
Source: JMIR Med Educ. 2018 Feb 26;4(1):e5. doi: 10.2196/mededu.7719 (PMC5847818; doi:10.2196/mededu.7719)
Supplement: Multimedia Appendix 4 [file mededu_v4i1e5_app4.pdf]

**Multimedia Appendix 4.** Clinical email update sent to staff at the comparator sites

"Dear health professional (nurse, doctor, pharmacist),

There have been some **important updates** to the adult guidelines for dosing, administration and monitoring of intravenous **vancomycin**, commonly used for the treatment and prevention of infections caused by MRSA.

Please see below for the key points:

**DOSING**

A **loading dose** is recommended, particularly in patients with serious infections who are critically ill. A dose of 25-30 mg/kg is appropriate in most situations.

For **maintenance doses** of vancomycin in an average weight patient (70kg):

| Creatinine clearance (mL/min) | Starting maintenance dosage | Timing of trough plasma level |
|---|---|---|
| more than 90 | 1.5 g 12-hourly | before the fourth dose |
| 60 to 90 | 1 g 12-hourly | before the fourth dose |
| 20 to less than 60 | 1 g 24-hourly | before the third dose |
| less than 20 | 1 g 48-hourly | 48 hours after the first dose |

- For intermittent dosing of vancomycin, an appropriate maintenance dose is 15-20 mg/kg (actual bodyweight). Use the Cockcroft-Gault formula or Web-based calculator to approximate creatinine clearance.

**ADMINISTRATION**

Vancomycin should be administered by slow infusion at a rate of **10 mg/min.**

**MONITORING**

- The recommended trough level for vancomycin is **15 to 20 mg/L** for most infections.

- Before interpreting the result, check that the timing of the trough sample was appropriate (i.e. before the last dose was given). In patients receiving vancomycin 12hrly, do not wait for the trough concentration result before giving the next scheduled dose.

- Adjustment of vancomycin dosage in adults:

| Trough plasma level | Dosage adjustment |
|---|---|
| less than 10 mg/L | Increase dosage by adjusting either the dose or the dose interval. |
| 10 to 14 mg/L | For patients with uncomplicated infection who are clinically improving, maintain current dosage. For patients with complicated infection, increase dosage by adjusting either the dose or the dose interval to achieve a trough concentration of 15 to 20 mg/L. |
| 15 to 20 mg/L | Maintain current dosage. |
| 21 to 25 mg/L | Maintain current dosage, or reduce dosage by adjusting either the dose or the dose interval, or withhold dose. |
| more than 25 mg/L | Withhold dose until trough concentration is less than 20 mg/L and seek expert advice. |

For any questions please contact …"

Clinical content based on *Therapeutic Guidelines: Antibiotic*, Version 15 [7].
